# A Comparative and Review Study on Shape and Stress Sensing of Flat/Curved Shell Geometries Using C^0^-Continuous Family of iFEM Elements

**DOI:** 10.3390/s20143808

**Published:** 2020-07-08

**Authors:** Mohammad Amin Abdollahzadeh, Adnan Kefal, Mehmet Yildiz

**Affiliations:** 1 Faculty of Engineering and Natural Sciences, Sabanci University, Tuzla, 34956 Istanbul, Turkey; abdollahzadeh@sabanciuniv.edu (M.A.A.); meyildiz@sabanciuniv.edu (M.Y.); 2 Integrated Manufacturing Technologies Research and Application Center, Sabanci University, Tuzla, 34956 Istanbul, Turkey; 3 Composite Technologies Center of Excellence, Istanbul Technology Development Zone, Sabanci University-Kordsa, Pendik, 34906 Istanbul, Turkey

**Keywords:** structural health monitoring, shape sensing, stress sensing, inverse finite element method, shell elements, strain sensor, damage detection

## Abstract

In this study, we methodologically compare and review the accuracy and performance of C^0^-continuous flat and curved inverse-shell elements (i.e., iMIN3, iQS4, and iCS8) for inverse finite element method (iFEM) in terms of shape, strain, and stress monitoring, and damage detection on various plane and curved geometries subjected to different loading and constraint conditions. For this purpose, four different benchmark problems are proposed, namely, a tapered plate, a quarter of a cylindrical shell, a stiffened curved plate, and a curved plate with a degraded material region in stiffness, representing a damage. The complexity of these test cases is increased systematically to reveal the advantages and shortcomings of the elements under different sensor density deployments. The reference displacement solutions and strain-sensor data used in the benchmark problems are established numerically, utilizing direct finite element analysis. After performing shape-, strain-, and stress-sensing analyses, the reference solutions are compared to the reconstructed solutions of iMIN3, iQS4, and iCS8 models. For plane geometries with sparse sensor configurations, these three elements provide rather close reconstructed-displacement fields with slightly more accurate stress sensing using iCS8 than when using iMIN3/iQS4. It is demonstrated on the curved geometry that the cross-diagonal meshing of a quadrilateral element pattern (e.g., leading to four iMIN3 elements) improves the accuracy of the displacement reconstruction as compared to a single-diagonal meshing strategy (e.g., two iMIN3 elements in a quad-shape element) utilizing iMIN3 element. Nevertheless, regardless of any geometry, sensor density, and meshing strategy, iQS4 has better shape and stress-sensing than iMIN3. As the complexity of the problem is elevated, the predictive capabilities of iCS8 element become obviously superior to that of flat inverse-shell elements (e.g., iMIN3 and iQS4) in terms of both shape sensing and damage detection. Comprehensively speaking, we envisage that the set of scrupulously selected test cases proposed herein can be reliable benchmarks for testing/validating/comparing for the features of newly developed inverse elements.

## 1. Introduction

Thin shell structures with monolithic/stiffened curved and flat geometries are commonly utilized in diverse engineering applications including ships and marine platforms, aerospace vehicles, and civil structures, among others. These structures should be strong enough to bear not only their own weights but also extreme environmental loads such as high wind pressure, catastrophic ocean waves and rainstorms without losing their structural integrity. These loads may result in material degradations due to the formation of cracks, voids, and stress intensification locations. In addition to the load-induced damages, the corrosive environmental conditions encourage the occurrence of additional failure modes including stress-corrosion cracking, fretting cracks, and thickness reductions due to the material erosion. Structures operating under these conditions eventually experience sudden failures and ruptures in their primary components, and therefore are rendered useless. Besides, sudden damages lead to economic loss, environmental pollution, and even may cause human casualties. To prevent such events and predict these undesirable damages in real time, a structural health monitoring (SHM) system with a reliable and robust displacement and stress-monitoring capabilities should be installed in the structures aboard [1,2,3].

SHM is a multidisciplinary technology that can provide a real-time estimation of strain and stress fields, overall structural deformations and damage positions through utilizing an ensemble of sensors discretely located on/in the structure acquiring physical/mechanical information such as strain, temperatures, acceleration and pressure. In recent decades, significant progress has been made in different forms of SHM technologies for various material and structural systems [4,5]. The SHM of simple and complex structural topologies (e.g., shell models with surface cracks [6,7]) made of isotropic materials (e.g., steel/aluminum structures [2,3,8]) or orthotropic materials (e.g., multilayered composite and sandwich structures [9,10]) are studied under static and dynamic loading conditions. Particularly, damage, delamination, and fatigue in laminated composite and foam-core sandwich structures were identified using embedded fiber optic sensors [11,12,13].

Apart from the conventional SHM approaches, a significant amount of attention has been paid to the real-time reconstruction of the structurally deformed shapes via strain sensors, i.e., a key technology for SHM systems, commonly referred to as “displacement monitoring” or “shape sensing”. Mathematically speaking, shape sensing is an inverse problem. For the solution of this inverse problem, various mathematical formulations and algorithms have been proposed and investigated experimentally and/or numerically for beam/plate/shell structures. In general, these algorithms can be classified into the following two main categories: (1) modal/analytical/curve-fitting approaches [14,15,16,17,18,19], and (2) inverse finite element method (iFEM) [20,21]. Davis et al. [14] used analytical trial functions for shape and vibration mode sensing in order to regenerate static-beam response using fiber Bragg grating (FBG) sensor data. The main drawback of their approach is the requirement of excessive numbers of strain sensors and trial functions to model complex modal shapes. Kang et al. and Bogert et al. [15,16] evaluated the dynamic response of a beam- or plate-like structure through computing modal coordinates using the strain–displacement relationship and discrete strain measurements obtained from surface-mounted FBG sensors. However, since the numbers of estimated mode shapes are restricted to the numbers of strain sensors, the accuracy of this approach may diminish for shape-sensing under dynamically complex loading conditions, thus limiting the generality of this approach. Kim and Cho [17] analytically approximated the deflection of a beam using a high-order polynomial function whose weights were found through curve-fitted experimental strains. Ko et al. [18] generalized Kim and Cho’s approach further and demonstrated its implantation to the shape-sensing of a wing-shaped beam model with airfoil cross-section subjected to bending deformations. However, the approaches [17,18] are unable to predict accurate torsional deformations due to the simplifications made in the kinematic relations utilized in the model. Chierichetti [19] introduced a non-linear numerical method called a load confluence algorithm (LCA) which requires the numerical estimation of force before reconstructing the dynamic response of a beam. Nevertheless, since the LCA approach first reconstructs the loads, the statistical complexity of the loading condition may lead to undesirable errors in the prediction of displacement field. Overall, the above-stated shape-sensing methods encounter difficulties in dealing with structures with complex geometries and boundary conditions, and hence cannot be easily generalized for shape sensing of any structures.

Among these inverse-methods, the iFEM methodology has been demonstrated to be the most general shape-sensing algorithm because of its mathematical attributes, i.e., the utilization of a least-squares variational principle based on experimental and numerical strain–displacement relationships [20,21]. In other words, this variational principle relies on the minimization of the squared norm errors between experimentally measured and numerically evaluated section strains. For a given structure, the experimental section strains can be calculated by using tri-axial surface strains obtained from strain rosettes that can be in the form of FBGs or conventional strain gauges. The numerical counterparts of such section strains can also be readily established for an infinitesimally small domain, i.e., an inverse finite element. The minimization of the iFEM least-squares functional with respect to the unknown displacements enables one to cast the final set of equations in a matrix-vector form, which can be solved through imposing problem-specific constraint boundary conditions into the final equations. Such a solution first reveals the full-field structural displacements, leading to shape-sensing in three-dimensional space. In the post-processing stage, the solution of the displacement field can be transformed into the strains via strain–displacement relations. Finally, the constitutive relationships of a given material can be used to evaluate the individual stress components, leading to stress monitoring in the framework of iFEM methodology. To recapitulate, the potential advantages of an iFEM algorithm can be stated concisely as: (1) the independence from the external loads; (2) the suitability for modelling the complex structural geometries with intricate constraints; (3) the utilization of only discrete strain measurements without needing material information; (4) the applicability to real-time analysis [2,3].

The iFEM methodology was first introduced for plate structures based on the kinematic relationships of the first-order shear-deformation theory (FSDT) [21]. Then, its original variational principle was adapted to the Timoshenko beam theory for the shape-sensing of beam structures [22], which is validated against experimental studies [23,24,25]. Subsequently, the FSDT-based iFEM formulation was extended to the utilization of the zigzag kinematics of refined zigzag theory (RZT) [26] for the shape-sensing of composite structures [27,28,29]. Since the iFEM formulation requires the discretization of the structural domain with inverse elements, various inverse beam-, plate- and shell elements were developed. The FSDT-based iFEM elements available in the literature include a three-node triangular inverse-shell element (iMIN3) [30], a four-node quadrilateral inverse-shell element (iQS4) [31], and an eight-node curved quadrilateral inverse-shell element (iCS8) [32]. All these elements possess the C^0^-continious shape functions. In particular, both iMIN3 and iQS4 utilize the first-order anisoparametric shape functions developed for triangle [33] and quadrilateral [34] finite elements, respectively. Such anisoparametric functions were obtained through imposing Tessler-Dong [35] constant shear-edge conditions along the element boundaries. On the other hand, the iCS8 element employs the Lagrangian serendipity (isoparametric) shape functions of an eight-node quadrilateral element [36]. To date, the iMIN3 element was extensively scrutinized for the numerical and experimental shape-sensing applications of plate structures with and without large deformations [37,38]. Besides this, the iQS4 was numerically demonstrated to be a practical element in modelling and obtaining accurate sensor configurations for the displacement monitoring of complex marine structures such as bulk carriers, chemical tankers, and offshore structures, among others [39,40,41,42]. As for the iCS8 element, its potential benefit for the shape-sensing of curved geometries with a low number of sensor measurements was demonstrated on curved marine structures [43]. Although iQS4 was demonstrated to be applicable accurately for the shape-sensing of slender composite structures [44], it is prudent to state that there are other iFEM elements mainly implemented for thick sandwich structures, such as RZT-based inverse-plate/shell elements [27,28,45].

To the best of the author’s knowledge, none of the previous research on the iFEM includes an extensive comparison of the C^0^-continuous inverse-shell elements (i.e., iMIN3, iQS4, iCS8) in terms of their efficiency and accuracy for the shape-sensing of various geometries with sparse and/or dense sensor deployments. Moreover, earlier studies on these inverse-shell elements [30,31,32] do not include any systematic investigation into the validation of these elements through analyzing benchmark geometries ranging from simple to complex features with/without any material degradation. In this study, the aforementioned issues in the literature are addressed through performing various shape-sensing analyses of plane, curved, monolithic, and stiffened engineering geometries with these three iFEM shell elements. Therefore, the original contribution of this study to the existing state-of-the-art includes not only the comparison of three inverse-shell elements but also the provision benchmark solutions, which can be used for the development of application-specific new inverse-element types. Furthermore, the present study reviews and emphasizes the significance of selecting the proper inverse-shell element type for a problem-specific application through considering the complexity of geometrical features. 

Since the fundamental kinematic relations of these three inverse elements are implemented based on FSDT, the iFEM weighted-least-squares functional of each element contains the contributions of the membrane, bending, and transverse-shear section strains, thereby allowing for a quantitative comparison of these three elements. To do so, the same geometry is discretized, using the same number of elements, and the same path of sensor lines is followed with the same number of sensor measurements. In addition, different sparse sensor placement strategies are examined with the aim of achieving the most practical and economical number of sensors in the discretization domain. Overall, four different plane/curved geometries, e.g., flat, curved, stiffened curved and curved with material degradation, with different loading and constraint conditions, are analyzed, as in the benchmark cases. For the last benchmark, an eye-shaped domain in the center of the curved plate is modelled as a material with degenerated (damaged) mechanical properties. With this problem, the damage detection capabilities of the C^0^-continuous inverse-shell elements are also explored, based on the same damage-detection criterion [46,47], thus providing benchmark guidance for the selection of iFEM shell elements as a part of SHM.

## 2. The iFEM Formulation Based on FSDT

**Figure 1:**
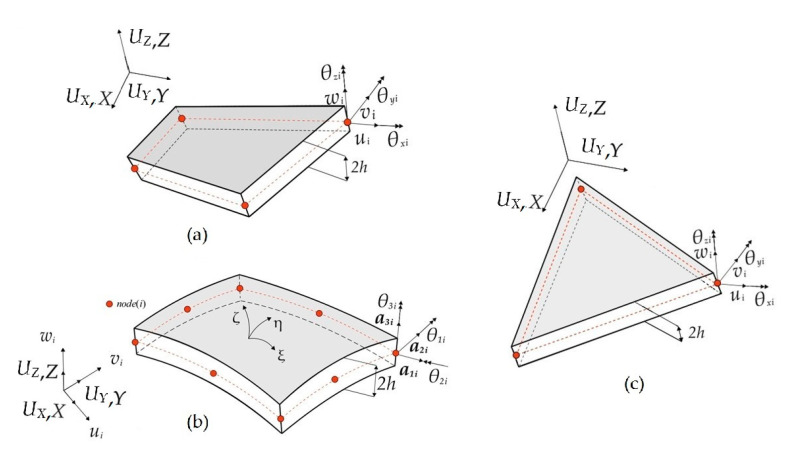
Geometries of (**a**) iQS4, (**b**) iCS8, and (**c**) iMIN3 inverse-shell elements with associated global and local coordinate systems as translational and rotational degrees of freedom (DOF).

The iMIN3, iQS4, and iCS8 inverse-shell elements are the three main iFEM discretization units used in the literature to perform shape-sensing of plate-like structural components in engineering applications. The displacement definitions of all these elements are based on the kinematic relations of the FSDT. The iMIN3 is the first inverse-shell element introduced in the literature [30] and possesses a flat triangular geometry containing three-nodes with six degrees of freedom (DOF) per each node, as shown in Figure 1. Although this inverse element has shown to be a good candidate for model structures with arbitrary shapes, the discretization of a complex geometry may require a higher number of elements as compared to quad-shaped inverse-elements, hence requiring the utilization of more strain sensors. 

Aa an alternative to the iMIN3 element, one may prefer to use a four-node quadrilateral inverse-shell element (iQS4) [31] for the discretization of a complex geometry with a lower number of elements. Similar to the iMIN3 element, the iQS4 also has a flat geometry and each node contains six DOF, i.e., the positive translational and rotational directions depicted in Figure 1. For modelling complex geometries, the main advantage of using both iMIN3 and iQS4 is associated with the inclusion of the drilling rotation, which prevents the singularity issues when discretizing a built-up structural geometry and enables a better shape-sensing capability for membrane deformations.

Apart from these elements, an eight-node inverse curved-shell element, iCS8, was introduced to tackle the shape-sensing of curved structural members [32]. Unlike iMIN3 and iQS4 elements, the in-plane geometry of this new element is constructed using curvilinear coordinates of ξ and η, as shown in Figure 1b, whereas the thickness coordinate, ζ, is rectangular to the in-plane coordinates along the thickness of the element. Each node of the iCS8 contains three translational DOFs, defined according to a global rectangular Cartesian coordinate system. Moreover, there are two local rotational DOFs employed to construct the kinematics relationships suitable for FSDT. Furthermore, an artificial drilling rotation is included in the displacement relations to avoid singular solutions when modelling stiffened curved geometries. The main advantage of iCS8 can be attributed to its geometrically conforming nature to structures with a lower mesh density. Overall, the iMIN3 and iQS4 elements utilize second-order anisoparametric shape functions to interpolate the translational kinematic variables, whereas the iCS8 element uses second-order (Lagrangian serendipity) isoparametric shape functions to approximate the displacement components. The detailed mathematical definitions corresponding to shape functions, displacement approximations, and displacement–strain relationships about the inverse elements studied herein can be found in [30,31,32].

Once the displacement field of each individual element is determined, the strain components at any point of the plate/shell domain can be analytically calculated in terms of the nodal displacement vector, $u^{e}$, of an element as

(1a) $$\left[\begin{array}{cc} \varepsilon & \gamma \end{array}\right]\equiv \left[\begin{array}{cc} \varepsilon (u^{e}) & \gamma (u^{e}) \end{array}\right]=\left[\begin{array}{cc} B^{\varepsilon }u^{e} & B^{\gamma }u^{e} \end{array}\right]$$

(1b) $$u^{e}={\left[\begin{array}{cccc} u_{1}^{e} & u_{2}^{e} & \cdots & u_{n}^{e} \end{array}\right]}^{T}$$

where the ε and γ vectors represent the in-plane and transverse-shear strains, respectively. In Equation (1b), the subscript n denotes the number of nodes of an individual element, e.g., iMIN3, iQS4, and iCS8. Moreover, the $B^{\varepsilon }$ and $B^{\gamma }$ matrices contain the derivative of the shape functions associated with the displacements corresponding to membrane-bending and transverse-shear deformations, respectively. Note that, for flat inverse-elements, coupled membrane-bending responses can easily be described as the sum of the membrane and bending section strains as

(2) $$\varepsilon (u^{e})=e(u^{e})+z\kappa (u^{e})=(B^{m}+zB^{b})u^{e}=B^{\varepsilon }u^{e}$$

where the vectors $e(u^{e})$ and $\kappa (u^{e})$ represent the membrane strains and bending curvatures in a given order. The strain–displacement relationship matrices, $B^{m}$ and $B^{b}$, corresponding to these individual section strains, were explicitly provided for flat iMIN3 and iQS4 elements in [30,31]. Such a decoupled form of strain definition (i.e., Equation (2)) may require cumbrous mathematical partitions for the iCS8. The explicit form of $B^{\varepsilon }$ matrix for the curved iCS8 element can be found in reference [32].

**Figure 2:**
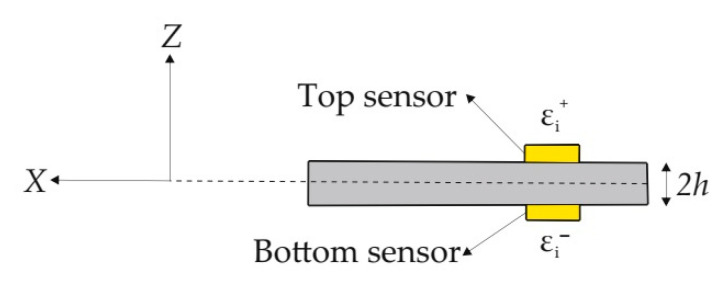
Experimental strain data collected from the top and bottom surfaces of a plate structure.

The main input of the iFEM methodology is the experimental strain measurements collected from the on-board strain sensors located at discrete positions of a given plate/shell-like structure, as shown in Figure 2. Consider that the $\varepsilon_{i}^{+}$ and $\varepsilon_{i}^{-}$ experimental surface strains are collected from the top (z=+h) and bottom (z=−h) thickness coordinates of different in-plane positions, $x_{i}\;(i=1,2,\ldots ,n_{s})$. Herein, the ‘+’ and ‘−’ superscripts are used to denote top and bottom surfaces, respectively, and the $n_{s}$ indicates the number of strain rosettes available on either bounding surface. Using these in situ surface strains, the experimental counterparts of the numerical strain data can be calculated as

(3) $$\left[\begin{array}{cc} e_{i}^{\varepsilon } & \kappa_{i}^{\varepsilon } \end{array}\right]\equiv \left[\begin{array}{cc} \frac{1}{2}(\varepsilon_{i}^{+}+\varepsilon_{i}^{-}) & \frac{1}{2h}(\varepsilon_{i}^{+}-\varepsilon_{i}^{-}) \end{array}\right]\;(i=1,2,\ldots ,n_{s})$$

where the $e_{i}^{\varepsilon }$ and $\kappa_{i}^{\varepsilon }$ vectors represent the experimental counterparts of the membrane, $e(u^{e})$, and bending, $\kappa (u^{e})$, section strains given in Equation (2), respectively. Such experimental strains can be combined together to obtain a coupled membrane-bending section strain experimentally as

(4) $$\varepsilon_{i}^{\varepsilon }=e_{i}^{\varepsilon }+z\kappa_{i}^{\varepsilon }\equiv e_{i}^{\varepsilon }+\zeta h\kappa_{i}^{\varepsilon }\;(i=1,2,\ldots ,n_{s})$$

where the $\varepsilon_{i}^{\varepsilon }$ vector represents the experimental counterpart of the $\varepsilon (u^{e})$ strains measured at the positions of $x_{i}$, i.e., continuously along the thickness coordinates, ζ∈[−1,1],  z∈[−h,h]. Experimental transverse shear strains, $\gamma^{\varepsilon }$, cannot directly be obtained from the $\varepsilon_{i}^{+}$ and $\varepsilon_{i}^{-}$ measurements. Nevertheless, when performing shape-sensing for slender structures, the contributions of transverse-shear strains to bending deformations can be safely omitted.

Based on experimentally measured and the numerically calculated section strains, the weighted least-squares functional of iFEM methodology can be defined for shape-sensing simulations by using iMIN3, iQS4, and iCS8 elements as

(5) $$\Phi (u^{e})=\frac{1}{V}\int_{V}\left(w_{\varepsilon }{\left\|\varepsilon (u^{e})-\varepsilon^{\varepsilon }\right\|}^{2}+w_{\gamma }{\left\|\gamma (u^{e})-\gamma^{\varepsilon }\right\|}^{2}\right)dV$$

where the V parameter represents the volume of an individual inverse-shell element, $w_{\varepsilon }$ and $w_{\gamma }$ are the weighting coefficients associated with the in-plane and transverse-shear strains. These coefficients can be set to unity, $w_{\varepsilon }=w_{\gamma }=1$, if both experimental measurements, $\varepsilon^{\varepsilon }$ and $\gamma^{\varepsilon }$, exist within a given inverse element. Otherwise, they should be set to a small number compared to unity, such as $w_{\varepsilon }=w_{\gamma }=10^{-4}$, in case of missing experimental section strains in the element domain. More details on the weighting coefficient strategies can be found in [2]. Minimizing the $\Phi (u^{e})$ functional with respect to the unknown $u^{e}$ displacements of an inverse element, the compact form of the final equation set can be obtained as

(6) $$\frac{\partial \;\Phi (u^{e})}{\partial \;u^{e}}=k^{e}u^{e}-f^{e}=0\Rightarrow k^{e}u^{e}=f^{e}$$

where the $k^{e}$ and $f^{e}$ are local analytical shape matrix and local experimental shape vector in the given order. The explicit forms of these quantities for iMIN3, iQS4, and iCS8 elements were provided in [30,31,32]. These local equation given in Equation (6) can be transformed into a global Coordinate system by using an appropriate transformation matrix, $T^{e}$, and then can be assembled for a given discretization composed of $N_{el}$ number of inverse element as

(7a) KU=F

(7b) $$K=\overset{N_{el}}{\underset{e=1}{\cup }}\left[{\left(T^{e}\right)}^{T}k^{e}T^{e}\right],\;F=\overset{N_{el}}{\underset{e=1}{\cup }}\left[{\left(T^{e}\right)}^{T}f^{e}\right],\;U=\overset{N_{el}}{\underset{e=1}{\cup }}\left[{\left(T^{e}\right)}^{T}u^{e}\right]$$

where the $\overset{}{\underset{}{\cup }}$ operator represents the classical finite element assembly process, and the K, U, and F denote the global shape matrix, displacement vector and experimental shape vector, respectively. The solution of the Equation (7a) is suitable for real-time monitoring process since the F vector is the only parameter that requires an update during each strain-data-acquisition in real time. At the final step, the problem-specific displacement constraints can be imposed on the Equation (7a), thus obtaining a reduced form of global equations that can be solved through an inversion/factorization process as

(8) $$K_{R}U_{R}=F_{R}\;\Rightarrow \;U_{R}=K_{R}{}^{-1}F_{R}$$

Hence, the overall deformed shape, i.e., the unknowns of the shape-sensing problem, can be easily reconstructed by combining the constraints conditions with the reduced displacement vector. After that, equivalent structural states constituting total displacement, $U_{T}$, and von Mises strain and stress, $\varepsilon_{vm}$ and $\sigma_{vm}$, can be readily calculated as

(9a) $$U_{T}=\sqrt{U_{X}{}^{2}+U_{Y}{}^{2}+U_{Z}{}^{2}}$$

(9b) $$\varepsilon_{vm}=\frac{\sqrt{2}}{3}\sqrt{{\left(\varepsilon_{1}-\varepsilon_{2}\right)}^{2}+{\left(\varepsilon_{2}-\varepsilon_{3}\right)}^{2}+{\left(\varepsilon_{1}-\varepsilon_{3}\right)}^{2}}$$

(9c) $$\sigma_{vm}=\frac{1}{\sqrt{2}}\sqrt{{\left(\sigma_{1}-\sigma_{2}\right)}^{2}+{\left(\sigma_{2}-\sigma_{3}\right)}^{2}+{\left(\sigma_{1}-\sigma_{3}\right)}^{2}}$$

where the $U_{X}$, $U_{Y}$, $U_{Z}$ symbols represent the displacements along global axes, and the $\varepsilon_{1}$, $\varepsilon_{2}$, $\varepsilon_{3}$ and $\sigma_{1}$, $\sigma_{2}$, $\sigma_{3}$ symbols indicate the principal strains and stresses, respectively. The accuracy of the individual iFEM elements studied herein is assessed by calculating the percent difference between the reconstructed structural responses and their reference solutions (i.e., obtained from high-fidelity FEM analysis) as

(10) $$P\mathrm{er}centDifference(\%)=\left|\frac{\delta_{iFEM}-\delta_{FEM}}{\delta_{FEM}}\right|\times 100$$

where the δ parameter can correspond to either total displacement or von Mises strain/stress.

## 3. Numerical Examples

In this study, we have solved four scrupulously selected benchmark cases that enable one to reveal the advantages and disadvantageous of the three inverse-shell elements under the same sensor configurations. The complexity of the benchmark cases is increased gradually from the plate-like to the curved stiffened shell structures, and finally covering a curved-shell with a damaged region. These geometrical features cover a wide range of practical engineering applications such as monolithic and stiffened flat/curved plate-/shell-like structural components. The cases studied herein include a tapered (wing-shape) plate, a curved shell, a stiffened curved structure and a curved shell with an imperfection (damage), in the given order. In addition, the boundary conditions in each test case are set in a prudent way to experience various types of deformations including in-plane stretching, bending, torsion, and their coupled responses. Since the order or complexity increases from the first to the last test case, these problems constitute a reliable set of benchmark cases for assessments of new inverse elements.

### 3.1. A Tapered Plate

**Figure 3:**
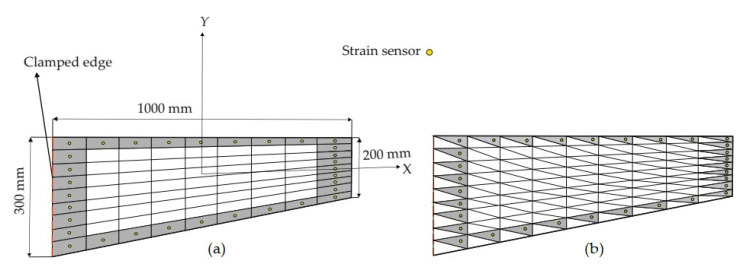
(**a**) Dimensions of the tapered plate with sensor placement model on iQS4/iCS8 mesh and (**b**) iMIN3 mesh.

A tapered plate with the dimensions, discretization, and sensor configurations shown in Figure 3 is analyzed based on three different iFEM elements. The plate has a thickness of 10 mm and is made of steel with the elastic modulus of 210 GPa and Poisson’s ratio of 0.3. The left edge of the plate is fully clamped against translational and rotational displacements and a body force of 63.765 kN/m^3^ is applied on the plate domain. As depicted in Figure 3a, the edges of the domain of interest are composed of nine subdivisions, leading to 81 quad-shaped (iQS4/iCS8) elements. To construct the tria-shaped (iMIN3) subdomains, the 81 quadrilateral elements were divided into two triangular elements with a single diagonal (Figure 3b). Given that the plate is made of an isotropic material, and its geometry is symmetrical with respect to the reference mid-plane, the strains at the top/bottom surface of the same in-plane positions will possess the same absolute values when subjected to the pure bending/torsional loads. Therefore, the sensor can be located at the top surface of the plate only. Here, the sensors are placed along the edgewise iFEM elements of the domain, resulting in 32 elements being instrumented with sensors (Figure 3). Since the expected deformations of the plate are bending dominated, the normal strain along the x-axis would vary significantly from the clamped region to the tip. Moreover, due to the tapered shape of the plate, there is an expected torsional deformation, which may cause different strain values and variations along the top and bottom edges of the plate. Furthermore, this torsional deformation also causes variations of the normal strain along the y-axis and the maximum values of such strain distribution are likely to be observed at the boundaries of the domain (i.e., the clamped and tip region). As depicted in Figure 3, we distribute the sensors at the boundaries along both longitudinal and transverse coordinates of the domain. In other words, the sensor placement model conforms to the perimeter of the domain of the interest, thereby enabling one to perform coupled bending-torsional deformation monitoring.

Suitable weighting coefficients (i.e., $w_{\varepsilon }$ and $w_{\gamma }$) should be defined to ensure the strain-interpolation continuity over the iFEM discretization. In the case of a strainless element (experimental strains, either $\varepsilon^{\varepsilon }$ or $\gamma^{\varepsilon }$, are missing), the weighting coefficients of the element can be set to a small value compared to unity, which can be defined in the range of 10^−3^ – 10^−5^. In this test case, the variation of the maximum iFEM-reconstructed displacement converges to a stable value as the small weighting coefficient decreases down to 10^−3^, and any further decrease in weight will not affect the convergence of displacement results. Considering these strategies, for the elements attached with the sensors, the weighting coefficients associated with the membrane and bending sections strains are assigned to unity. Since the span-to-thickness ratio of the present plate is large enough, the transverse-shear section strains will be negligibly small ($\gamma^{\varepsilon }\approx 0$), as in the case of a thin plate model. Therefore, the weighting coefficients for the transverse-shear sections strains are set to $10^{-3}$ and no experimental transverse-shear strains is used in the iFEM analysis. In addition, for the elements without any sensors, their weighting coefficients of the membrane-bending strains are assigned to a small value (i.e.,$10^{-3}$) as well, in order to preserve the strain–interpolation connectivity between iFEM elements with sensors. First, a direct FEM analysis with a fine mesh consisting of 729 elements is performed to obtain accurate displacement results that can be used as a reference solution for the iFEM analysis. Moreover, the strain values obtained from the high-fidelity FEM analyses are used to simulate the experimental in situ strain measurements at the center of the iFEM elements. Subsequently, three different iFEM analyses are performed by using the iCS8, iQS4, and iMIN3 elements.

**Figure 4:**
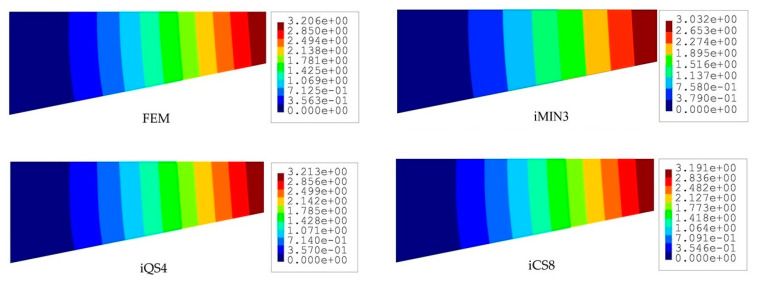
Contours of total displacement [mm] for the tapered plate.

**Figure 5:**
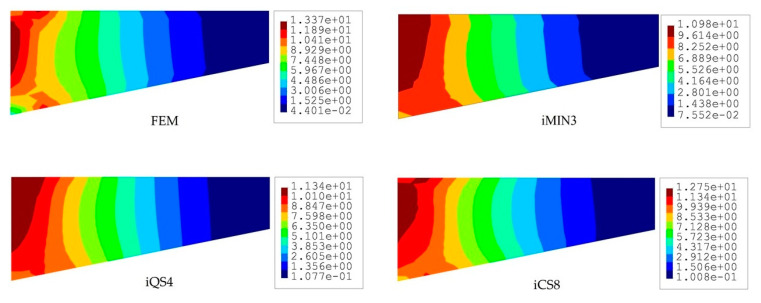
Contours of von Mises stress [MPa] for the tapered plate.

The iFEM results are compared in terms of displacements and stresses with respect to the direct FEM analysis, as given in Figure 4 and Figure 5. Here, one can clearly observe from Figure 4 that all iFEM elements generate nearly the identical displacement contours indistinguishable from those of FEM throughout the plate domain. However, the percent differences between iMIN3, iQS4, iCS8 and reference solutions for the maximum total displacements are approximately 5.42%, 0.21%, 0.53%, respectively, therefore the maximum displacements obtained from iQS4 and iCS8 are closer to the maximum reference displacements than the iMIN3. This observation can be attributed to two important physical/mathematical aspects: (1) the total number of sensors per total number of edgewise iFEM elements, and (2) shape function construction. As for the iMIN3 sensor placement model, fifty percent of the edgewise elements are populated with sensors, while all edgewise elements in the iQS4 and iCS8 models possess one-to-one sensor placement. Referring to the shape function construction, anisoparametric shape functions of the iMIN3 element use the area-parametric coordinates of a triangle, whereas the iQS4 element’s anisoparametric shape functions utilize the bilinear isoparametric mapping functions. Since the area-parametric coordinates have a less accurate interpolation capability than the Lagrangian mapping functions, the displacement approximation achieved by iQS4 and iCS8 elements is superior to that of iMIN3. To recapitulate, although iQS4 and iCS8 have inherently different shape functions, they perform in the same manner in terms of displacement reconstruction on a flat plate.

To compare the performance of these three iFEM elements further, we perform a posteriori calculation and obtain the von Mises stress distributions across the plate domain as shown in Figure 5. Although all three elements produce similar variations in stress contours along the length of the tapered plate, the iCS8 element renders a better reconstruction of the von Mises stresses in the vicinity of the clamped region as compared to the flat elements. The percent differences between reference solutions and iFEM elements (iMIN3, iQS4, and iCS8) for the maximum von Mises stress are about 17.87%, 15.33%, and 11.66% in the given order. This quantitative assessment bespeaks the higher order accuracy of the iCS8 elements for stress sensing, which is associated with the fact that strain distribution in the iCS8 element is not uniform along spatial coordinates and possesses a high-order polynomial with respect to the flat elements. This is further supported by the non-oscillatory and smooth stress contours obtained by the iCS8 element, which are almost identical to those of FEM analysis. 

### 3.2. A Curved Plate

**Figure 6:**
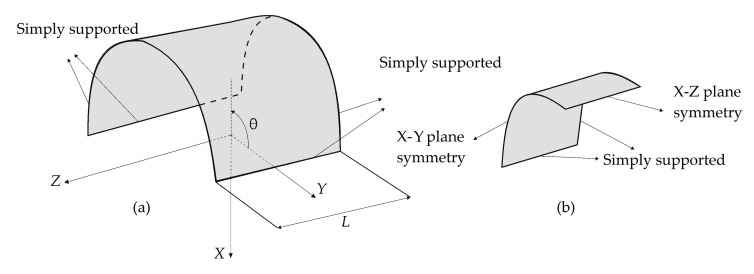
(**a**) Cylindrical shell; (**b**) one-fourth of the shell: curved plate with symmetric boundary conditions.

To compare the advantages and shortcomings of the three inverse elements for the displacement reconstruction of complex geometries, we considered a simply supported, cylindrical-thin shell structure (herein referred to as “curved plate”). The curved plate has a radius, length, and thickness of 100, 120, and 2 mm, respectively. The material properties of the curved plate are as same as the previous example. As illustrated in Figure 6a, four edges of the plate are simply supported. Moreover, the top surface of the plate is subjected to a sinusoidal pressure of the form, $q(z,\theta )=q_{0}\sin(\theta )\cos(\pi z/L)$, with pressure magnitude of $q_{0}=5$ [MPa], where the z and θ coordinates are bounded by z∈[−L/2,L/2],   θ∈[0,π]. 

**Figure 7:**
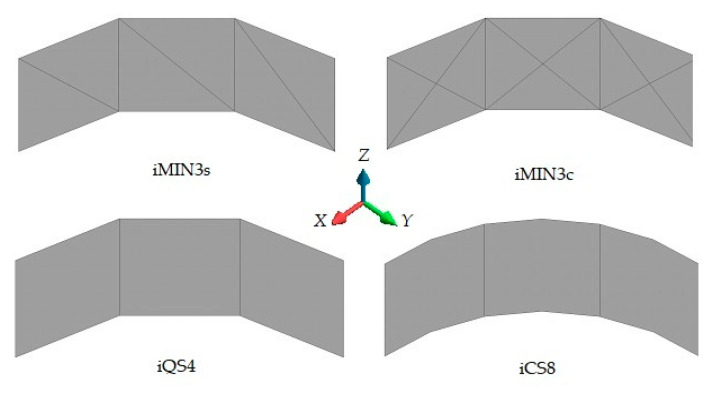
Mesh resolution of 3 × 1 for the curved plate using four different discretization strategies.

Due to the symmetrical nature of the computational domain (i.e., geometry, material, loading, and constraints), only one-fourth of the curved plate can be prudently analyzed using appropriate symmetry boundary conditions, as depicted in Figure 6b. To compare the convergence performance of the iFEM elements in terms of the mesh/sensor density, three different mesh resolutions (i.e., 3 × 1, 5 × 2, and 15 × 5 elements) ranging from coarse to fine mesh/sensor densities are constructed using iMIN3, iQS4, and iCS8 elements. For the sake of clarity, examples of coarse mesh resolutions (3 × 1) are illustrated in Figure 7. Being particular to the iMIN3 models, two different discretization schemes are proposed, namely single and cross-diagonal patterns (i.e., iMIN3s and iMIN3c), as shown in Figure 7. Here, tria-shape elements (i.e., offspring element) are generated by diagonally dividing the quadrilateral elements (i.e., parent element), thereby forming either two or four offspring.

To provide strain data as an input to the iFEM models as well as establish a reference solution for the curved plate, a direct FEM analysis was performed utilizing a sufficiently fine mesh composed of 105 × 45 quadrilateral elements with 29,256 DOF. The simulated data (i.e., representing the experimental data) is assigned to the geometric center of the each relevant iFEM element. For iMIN3s and iMIN3c models, the experimental strain value at the centroid of each specific parent inverse-element (iQS4/iCS8) is equally assigned to all offspring (iMIN3) elements. In this manner, the number of sensors for each tria- or quad-mesh becomes identical, so that there is no strainless element in the triangular configurations. 

**Figure 8:**
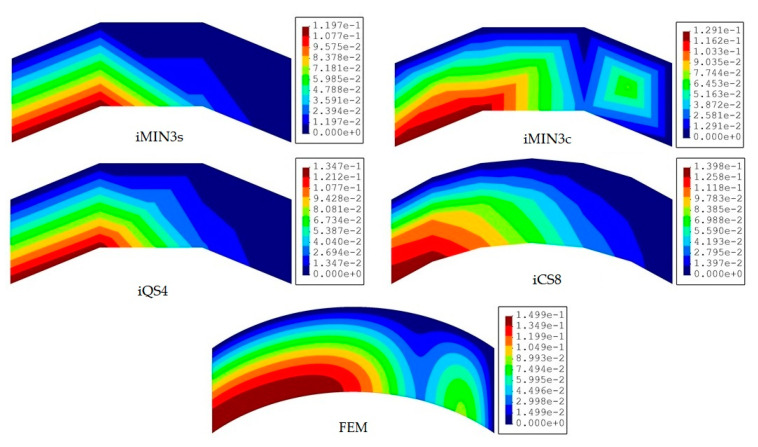
Contours of total displacement [mm] obtained from iFEM (coarse mesh) and high-fidelity FEM analyses for the curved plate.

**Figure 9:**
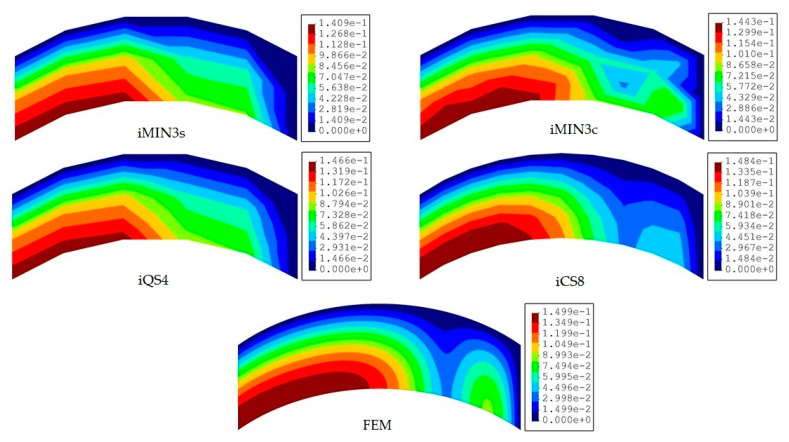
Contours of total displacement [mm] obtained from iFEM (moderate mesh) and high-fidelity FEM analyses for the curved plate.

**Figure 10:**
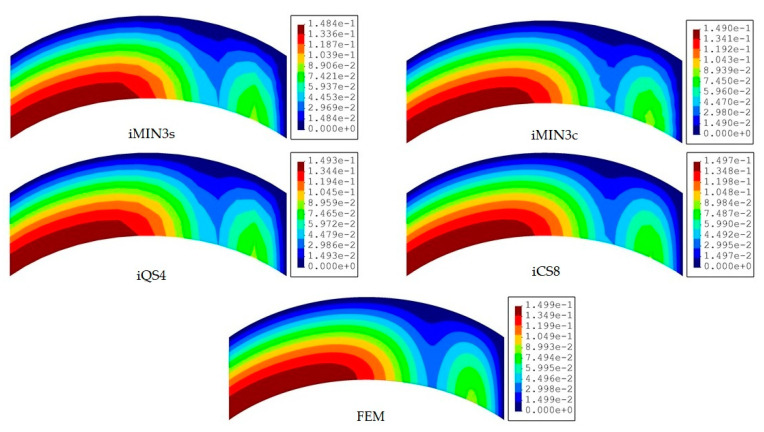
Contours of total displacement [mm] obtained from iFEM (fine mesh) and high-fidelity FEM analyses for the curved plate.

In this benchmark, the weighting constants associated with the membrane-bending and transverse-shear section strains are set to $w_{\varepsilon }=1$ and $w_{\gamma }=10^{-3}$ for all iFEM discretization, respectively. Afterward, the iFEM analysis for each mesh resolution of a given element type is performed and the results are presented and compared with respect to each other and the reference solution in terms of contours of total displacements in Figure 8, Figure 9 and Figure 10. One can see from Figure 8 that, at low mesh resolution, the iCS8 element generates displacement contours much more consistent with reference contours than those produced by iMIN3s/c and iQS4 models. Additionally, for moderate mesh resolution, the better shape-sensing capability of the curved elements in terms of accurate total displacement contours prevailed over the flat elements, as shown in Figure 9. Furthermore, as the mesh density increases (refer to Figure 9 and Figure 10), the difference between the results of all elements disappears and their displacement contours become almost indistinguishable from the reference solution.

**Figure 11:**
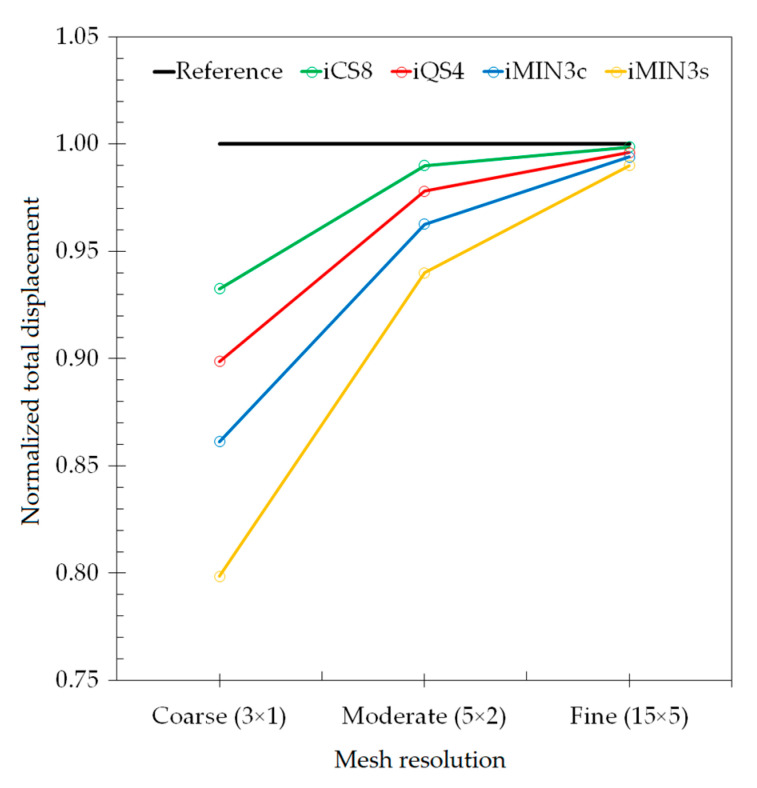
Comparison of normalized total displacements versus increasing mesh resolution for the curved plate.

To provide a quantitative comparison for all elements, the maximum values of the total displacements of each iFEM analysis are normalized with respect to that of FEM analysis, and these results are plotted versus the increasing mesh resolution in Figure 11. One can conclude that, for the modelling of a curved geometry, the curved element produces the lowest displacement errors with respect to the reference solution, even using the coarse discretization in the iFEM analysis. As elaborated on previously, the preeminent performance of iCS8 over all the other elements is associated with the high-order (serendipity) Lagrangian shape functions and the inclusion of a greater number of nodes, which enables a better physical representation of the geometry even in the case of low-mesh resolutions. It would be also interesting to compare the performance of the single and cross-diagonal mesh models of iMIN3 element. Accordingly, in Figure 11, the iMIN3s predicts maximum normalized displacements approximately 1.5 times more erroneous than the results of iMIN3c. Hence, even the same number of sensors is used in iMIN3s/c models; the iMIN3c entails a higher precision because of the central-node included in the parent quadrilateral elements, thereby enabling a better interpolation of the structural deformations in the parent domain. When the performance of the iQS4 and iMIN3c are compared in terms of displacement reconstruction, the iQS4 estimates displacements more accurately over the curved geometry, hence yielding the highest precision among the family of flat elements. Therefore, in the rest of the study, we only compare the performance of curved (iCS8) and more accurate flat (iQS4) elements.

### 3.3. A Stiffened Curved Plate

**Figure 12:**
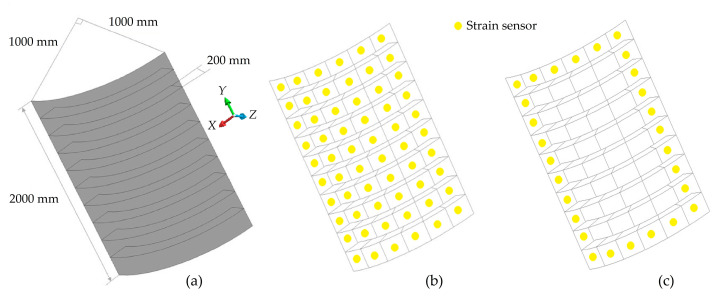
(**a**) Dimensions of the stiffened curved plate; (**b**) sparse and (**c**) very sparse sensor placement models.

Previous numerical examples have proven that the iMIN3s/c meshing strategies are not as accurate as other meshing methods using flat/curved inverse elements (namely, iCS8 and iQS4) in terms of reconstructing structural deformations. Therefore, we eliminate the comparative assessments of iMIN3 element in the remaining test cases and herein continue with the implementation of iCS8 and iQS4 on a more complicated geometry, i.e., a stiffened curved plate with dimensions depicted in Figure 12a. It is expected that the strain/stress distribution should include severe and non-smooth variations due to the presence of stiffeners as well as the curvilinear geometry of the plate. Therefore, this benchmark problem lends itself to revealing the capabilities of two high-performance iFEM elements (iQS4 and iCS8) comparatively in terms of both shape and stress-sensing. Specifically, the curved plate has a length, radius, and uniform thickness of 2000, 1000, and 30 mm, respectively. The plate has equally spaced transverse stiffeners with a height and thickness of 200 and 30 mm. The plate and stiffeners are made of an isotropic (steel) material with an elastic modulus, Poisson’s ratio, and density of 200 GPa, 0.3, and 7800 kg/m^3^, in the given order. All edges of the plate are simply supported, allowing for rotational DOF and constraining translational DOF only. Here, the stiffened curved plate is subjected to the gravity of 9.81 m/s^2^, thus resulting in the body force of 7800 × 9.81 N/m^3^. 

As in the case of previous test cases, the forward structural analysis is first performed using a high-fidelity FEM model composed of 1026 elements to generate a reference solution as well as strain-sensor data, i.e., the input of the iFEM analysis. The present iFEM analyses are performed using a discretization composed of uniformly distributed 114 quad-shape inverse-elements, namely either an iCS8 or iQS4 element. For this test case, three types of sensor placement models, namely ‘full’, ‘sparse’, and ‘very sparse’ with the labels f, s, vs, are used on the same iFEM mesh resolution. 

In the full model, all elements include strain sensors with 114 × 2 total number of strain rosettes. As for the sparse sensor placement model, only inverse elements on the surface of the plate are assigned to the input strain data, thus requiring the usage of 60 × 2 strain rosettes. For the last sensor configuration (vs model), the total number of strain rosettes was reduced to 28 × 2, since only inverse elements along the edges of the curved plate are instrumented with sensors. For clarity, representative sensor-placement configurations (s and vs models) are illustrated in Figure 12b,c. All weighting constants for strainless elements (without experimental strain data) are set to $10^{-4}$, while they are defined as $w_{\varepsilon }=1$ and $w_{\gamma }=10^{-4}$ for all elements with strain rosettes in the different iFEM models.

**Figure 13:**
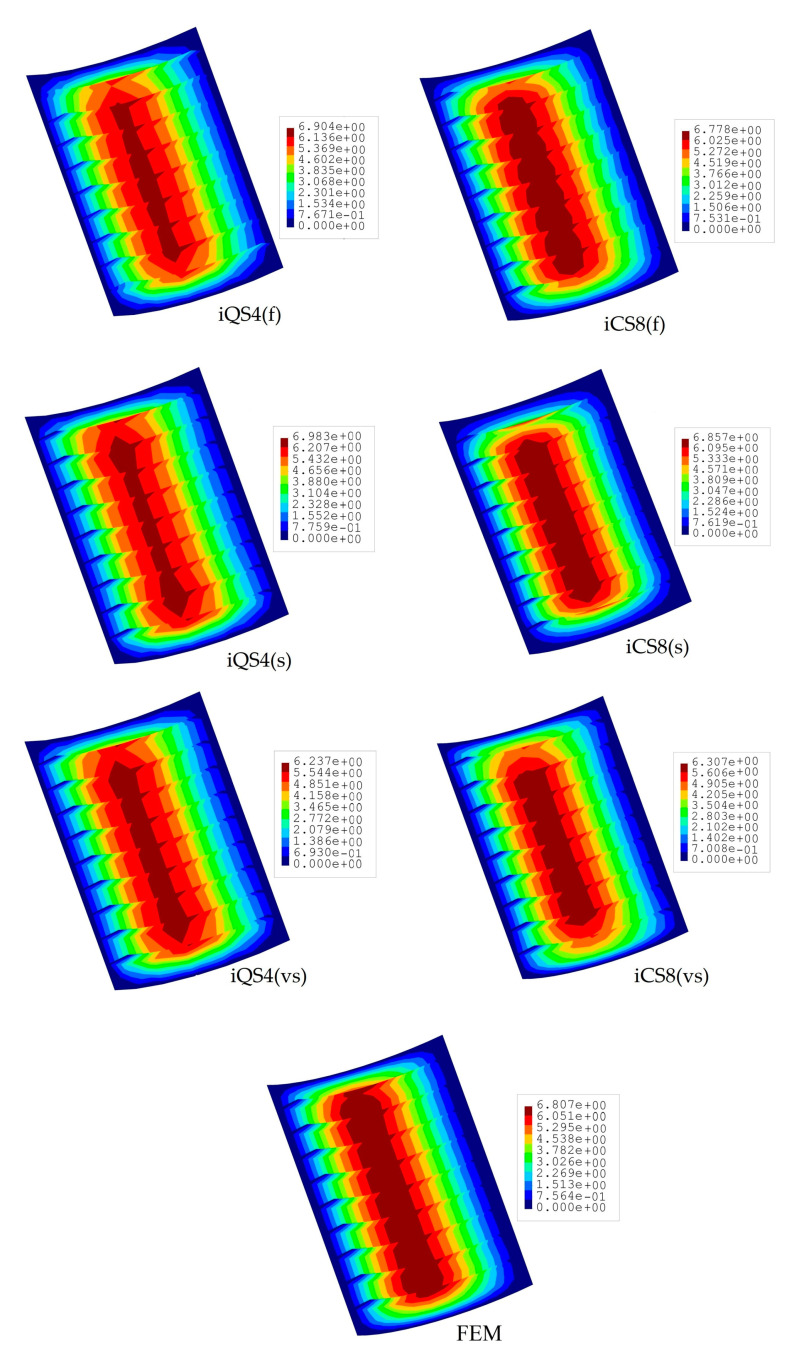
Contours of total displacement [mm] for stiffened curve plate.

In Figure 13, the total displacements contours of iQS4 and iCS8 models with different sensor density are compared to each other and with respect to the reference solution. As can be seen from the figure, the iQS4 produce a more localized maximum total displacement field along the centerline of the plate than those of iCS8 and the reference solution for all three sensor-placement configurations. This clearly indicates that the iCS8 element reconstructs total displacement field much better than iQS4 element for the given curved (complex) stiffened geometry. Quantitatively speaking, the percent differences for the maximum total displacements between iFEM and FEM analyses are calculated as (1.43%, 2.59%, 8.37%) and (0.43%, 0.73%, 7.35%) for the iQS4 and iCS8 (f, s, vs) models, respectively. At a higher sensor density, the iCS8 element predicts total displacement that have at least three times lower error than the iQS4 element, while this error reduces down to 1.15 at very sparse sensor deployment. Despite the closer error level between flat/curved elements for the vs model, it can be understood from the percent errors that as the sensor density increases, the predictive capability of the iCS8 becomes much better than the iQS4 element. Nonetheless, for any sensor density, iCS8 yields a superior shape-sensing than iQS4, which can be ascribed to the conforming nature of the quad-shape curved element. 

**Figure 14:**
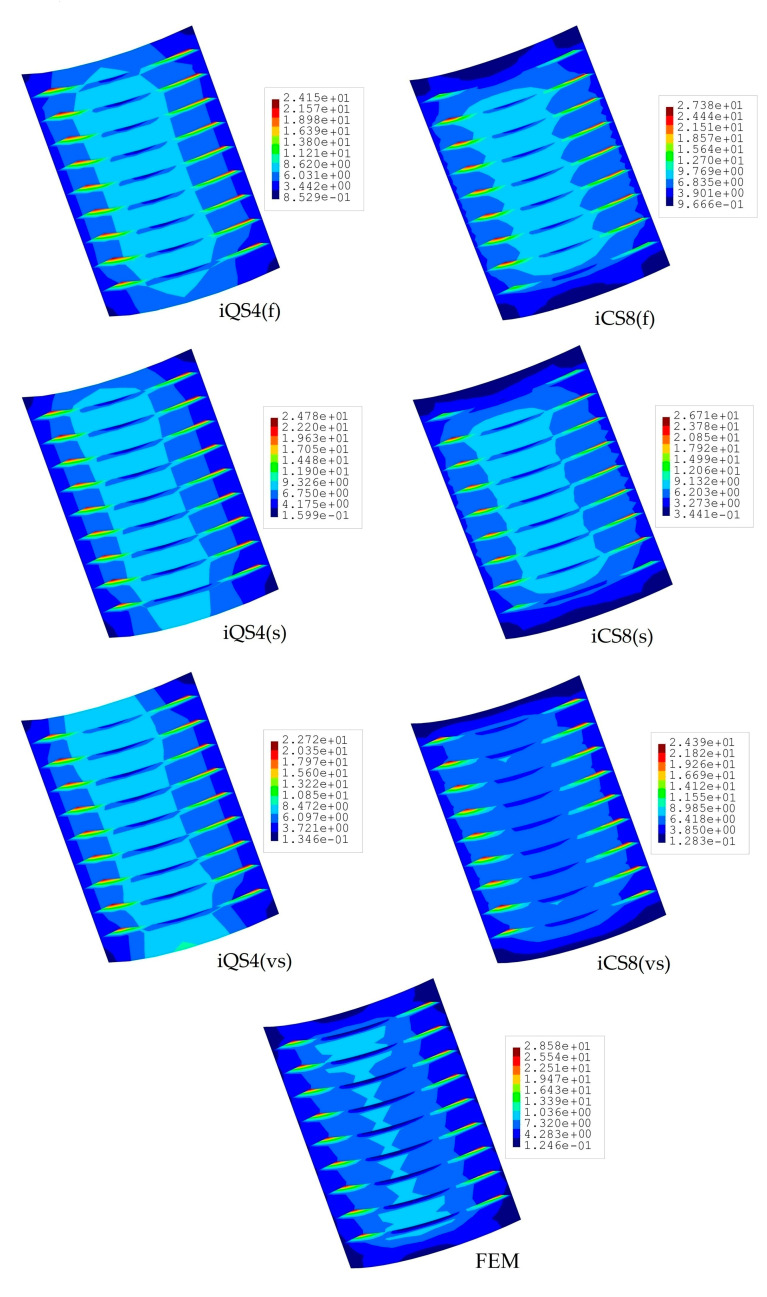
Contours of von Mises stress [MPa] for stiffened curved plate.

A similar comparison is also provided for the von Mises stress fields, as shown in Figure 14. Likewise, the percent differences for von Mises stress between iFEM and FEM analyses are 13.30%, 15.50%, and 20.50%, respectively, for iQS4 models with f, s, and vs sensor deployments. Correspondingly, they are 4.20%, 6.54%, and 14.94% for the iCS8 models. An important observation is that, although there are no sensors on the stiffeners in the s and vs sensor deployments, both the flat and curved elements of iFEM can predict accurate stress variations across stiffeners to an adequate degree. Overall, it is shown that the iCS8 element surpasses the predictive capability of the iQS4 in terms of not only the displacement field, but also the equivalent stress field.

All the benchmark cases were assessed again by the standard deviation measurement principle to further compare the accuracy and performance of iMIN3, iQS4, and iCS8 elements in terms of shape and stress sensing. The root–mean–square difference (RMSD) between iFEM and FEM results can be computed as

(11) $$RMSD=\sqrt{\frac{\sum_{i=1}^{N_{n}}{\left(\delta_{i}^{iFEM}-\delta_{i}^{FEM}\right)}^{2}}{N_{n}}}$$

where the $N_{n}$ term represents the total number of nodes in the global iFEM discretization and the δ parameter corresponds to either total displacement, $U_{T}$ or von Mises stress, $\sigma_{vm}$. As is inferred from Equation (11), the stability of the results obtained using different iFEM elements does not depend on a single node having the maximum displacement/stress value, since all nodes contribute to the increasing stability of the results in the standard deviation calculation.

The RMSD values computed for various test cases are listed in Table 1. These results clearly confirm the enhanced shape- and stress-sensing capability of iCS8 element against flat elements. Expectedly, the iQS4 element produces smaller RMSD values than iMIN3s or iMIN3c meshing strategies. For all cases (1–3), the displacement and stress results estimated using the iCS8 element have the lowest average deviation from reference FEM results, thereby proving its superiority against flat iFEM elements.

### 3.4. A Curved Plate with a Damaged Region

In many engineering structures, there are internal features in the form of holes, slots or cutouts due to the topological design or assembly requirement. These regions experience drastic strain/stress variation that needs to be closely monitored to ensure the structural integrity of the component under operational conditions. Such internal features can be modelled as a material region with degraded elastic constants or without any stiffness. Since these domains can be considered as pre-damaged locations, it will be very important and critical to accurately reconstruct the displacement and stress variation from the discrete sensor information collected at the far-field region of these pre-damaged positions. To this end, it is crucial to select effective iFEM elements for geometry-specific problems. Therefore, in this study, we have extended our benchmark cases further, such that the performances of two main iFEM elements are scrutinized comparatively in terms of their predictive capabilities for full-field shape and strain sensing of pre-damaged structure. Specifically, this test case focuses on a curved plate with an eye-shaped cutout, representing either an internal feature or a pre-damaged section of a structural component. The present investigation on the curved plate can also entail the performance evaluation of curved/flat elements for their usage in damage localization and detection sensitivity.

**Figure 15:**
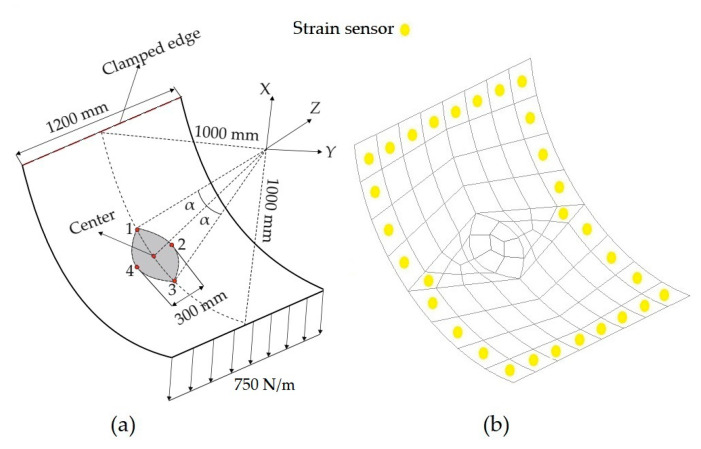
(**a**) Dimensions of the curved plate with a damaged region at the center; (**b**) sensor placement model for iCS8/iQS4 discretization.

The dimensions of the curved plate are provided in Figure 15a, where the eye-shaped region is symmetrically located with respect to the center of the curved plate with an angle of α=π/16. Two edges of this region are created by passing arcs from points of 1, 2, 3 and 1, 4, 3, in the given order. As depicted in Figure 15a, the top edge of the plate is fully clamped, with no rotational and translation DOF, and a line-distributed force of 750 N/m is applied at the bottom edge of the plate. Initially, the curved plate is considered to be free of any damage, representing the undamaged condition. Under the operating conditions, the plate is assumed to develop a defective eye-shaped region. Here, this region is particularly chosen to intensify the strain variation near the damaged region, thereby setting up a more challenging test case. To represent the intact and damaged (degraded) regions of the curved plate, two different elastic moduli are used, namely 210 GPa and 21 MPa for intact and defective regions, respectively, having the same Poisson’s ratio of 0.3. Similar to the previous test cases, a direct FEM analysis is used for simulating experimental sensor data and establishing reference solutions for both undamaged and damaged conditions of the plate. After FEM analyses, two iFEM analyses are performed using the discrete strain data collected from the undamaged and damaged conditions of the curved plate. As shown in Figure 15b, the iFEM model is composed of 88 quad-shape elements (representing either iCS8 or iQS4) and the edgewise elements accommodate the strain sensors, thereby resulting in 30 sensors in the model. During the iFEM analysis of the present geometry, the weighting constants are set to the values of the previous case study (corresponding to the sparse sensor configurations, namely either s or vs model). Once the displacements are reconstructed using iCS8 and iQS4 models, the von Mises strains are calculated for each node $\;i=1,2,\ldots ,N_{n}$ in the iFEM models. Then, to localize the damage, the damage indication factor, $\Omega_{i}$, is computed as [46,47]

(12) $$\Omega_{i}=\left|\varepsilon_{i}^{D}-\varepsilon_{i}^{U}\right|/\varepsilon_{\max}^{D}$$

where the $\varepsilon_{i}^{D}$ and $\varepsilon_{i}^{U}$ parameters represent the iFEM-based reconstructed or reference FEM von Mises strains at the nodes for the damaged and undamaged conditions of the curved plate, respectively, and the $\varepsilon_{\max}^{D}$ denotes the maximum value of the reconstructed von Mises strains for the damaged case. Finally, Equation (12) is used to estimate and compare the damage detection capability of iQS4 and iCS8 elements for the present test case. 

**Figure 16:**
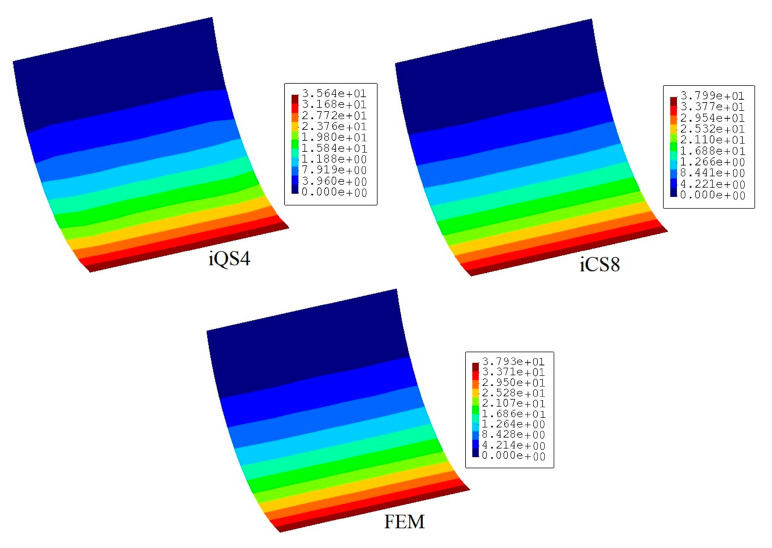
Contours of total displacement [mm] for the damaged condition of the curve plate.

**Figure 17:**
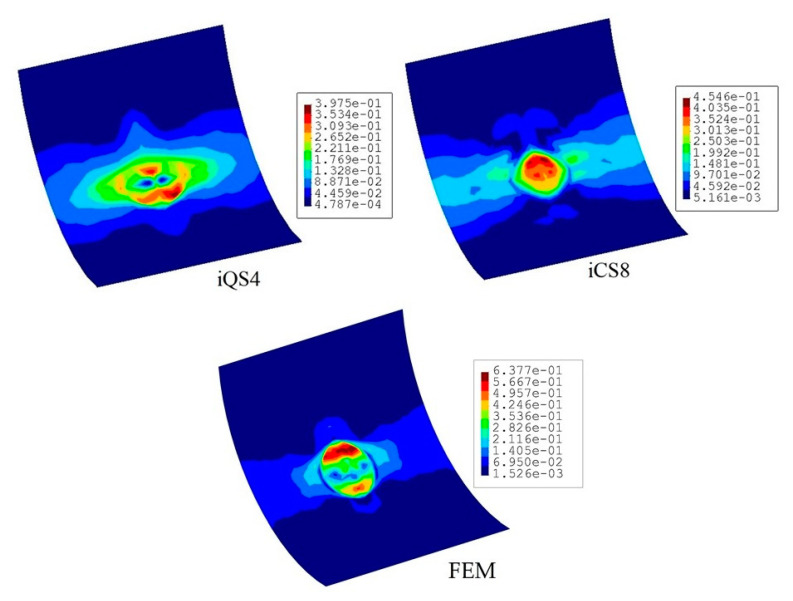
Contours of damage indication factor for the curved plate with a damaged region.

Figure 16 compares the reconstructed displacement of flat and curved elements with respect to the reference solution for the damaged configuration. As is evident from the comparison, the displacement contours do not imply the possible location of the damaged region. Nevertheless, according to the maximum displacement produced by iCS8 and iQS4, the result of the curved element is in a better agreement with the reference solution than the flat element. In numbers, the percent difference between iCS8 and reference solution for the maximum displacement is 0.15%, whereas it is 6.03% for the iQS4 element, thus confirming the higher precision of curved element for shape sensing of cylindrical geometry even using sparse sensor configuration. To reveal the damage localization capabilities of these elements, in Figure 17 the contours of damage indication factor are provided and plotted over the curved geometry. These damage factors are calculated using iFEM (iQS4 and iCS8 models) and FEM analysis. According to the comparison between iFEM and FEM damage predictions, it can be clearly seen from the damage contours in Figure 17 that the iCS8 element localizes the damage and detects its eye-shape better than the flat element. Hence, it can be concluded that iCS8 element offers a better shape sensing and structural health monitoring features for real-time monitoring of complex geometries.

## 4. Conclusions 

The shape-, strain-, and stress-sensing performance of iMIN3, iQS4, iCS8 elements are investigated and compared for various plate and shell structures by gradually increasing their geometrical complexity. These geometries include flat, curved, stiffened-curved, and a curved plate with a damaged region. For all iFEM elements, the weighted-least-squares functional is constructed according to the FSDT displacement kinematics. When the geometry of the structure is blade-like, as intuitively expected, there is no distinctive difference among all the iFEM-reconstructed displacement results with respect to the reference solution. A slightly better stress-sensing accuracy is observed for iCS8 element over the other elements for the flat geometry. This improved in the stress prediction of iCS8, which is attributed to its high-order serendipity shape functions allowing for an extra mid-node in the stress prediction. As the complexity of the geometry of the test cases increases (i.e., curved and stiffened-curved), it was proven that the predictive capability of iCS8 element becomes obviously superior to the flat elements for shape-sensing with a lower number of sensors and coarser mesh configuration. This is because the second-order isoparametric mapping functions of iCS8 allow for a smoother approximation of the curved geometries by using a lower number of elements. When the flat elements are compared to each other, it is observed that iQS4 yields a better displacement reconstruction than iMIN3 meshing strategies. These meshing configurations consist of single- and cross-diagonal divisions of a quad-element into tria-elements. Remarkably, the shape-sensing ability of iMIN3 can be enhanced if the cross-diagonal meshing strategy is utilized. Finally, the damage detection feature of the elements is explored on a curved plate with a geometrically complex damage region. Accordingly, the damage detection sensitivity of the curved element is observed to be much better than that of the flat element. Overall, all the test cases presented herein can serve as “*benchmark problems*” for the newly developed iFEM shell/plate elements. The optimization of sensor positions in an iFEM shape-sensing model (i.e., generated using C^0^-continious inverse plate/shell elements) can be considered as an extension of the current study in the future.

## Figures and Tables

**Table 1:** Root–mean–square difference (RMSD) results for $U_{T}$ displacement and $\sigma_{vm}$ stress of various test cases.

Structural Response	Case1			Case2 (Moderate)				Case3 (Sparse)	
	iMin3s	iQS4	iCS8	iMin3s	iMIN3c	iQS4	iCS8	iQS4	iCS8
$U_{T}$	0.116	0.014	0.009	0.0228	0.0221	0.0220	0.0219	0.530	0.411
$\sigma_{vm}$	0.508	0.116	0.067	-	-	-	-	3.620	2.772
